# Analysis of Patients with Glioblastoma Treated with Standard 6-Week Chemoradiation Followed by Temozolomide: Treatment Outcomes and Prognostic Factors

**DOI:** 10.3390/medicina61030376

**Published:** 2025-02-21

**Authors:** Sojung Lee, Myungsoo Kim

**Affiliations:** Department of Radiation Oncology, Incheon St. Mary’s Hospital, College of Medicine, The Catholic University of Korea, Seoul 06591, Republic of Korea; mskim0710@gmail.com

**Keywords:** glioblastoma, concurrent chemoradiation, inflammatory marker, prognostic factor

## Abstract

*Background and Objectives*: We aimed to investigate the treatment outcomes and prognostic factors of survival among patients with glioblastoma who underwent 6-week concurrent chemoradiation therapy (CCRT) followed by temozolomide (TMZ) with Stupp’s regimen in a single tertiary institution. *Materials and Methods*: Eighty patients with glioblastoma who underwent 6-week CCRT followed by TMZ between June 2010 and January 2024 were retrospectively investigated. A survival analysis was performed of factors such as age, O (6)-methylguanine-DNA methyltransferase promoter (MGMT) methylation, extent of resection, pre- and post-operative Karnofsky Performance Status, and inflammatory markers such as neutrophil-to-lymphocyte ratio, lymphocyte-to-monocyte ratio (LMR), and platelet-to-lymphocyte ratio. Post-operative inflammatory markers were assessed at 2–3 weeks post-operative before the initiation of CCRT. A subgroup analysis was performed of patients who underwent non-gross total resection (GTR). *Results*: The median progression-free survival (PFS) and overall survival (OS) of the entire cohort were 8.97 months and 19.0 months, respectively. Older age (≥65 years) and non-GTR status were adverse prognostic factors of PFS and OS. MGMT methylation is a favorable prognostic factor for PFS and OS. In the subgroup of patients who underwent non-GTR, MGMT methylation and post-operative LMR (<3.2/>3.2) were independent prognostic factors for PFS and OS. *Conclusions*: As with previous studies, older age, MGMT methylation, and extent of resection were independent prognostic factors for the survival of patients with glioblastoma who underwent standard treatment with Stupp’s regimen. MGMT methylation and post-operative LMR were significant prognostic factors for PFS and OS among patients who underwent non-GTR. The prognostic significance of post-operative inflammatory markers for treatment response and survival should be further validated in glioblastoma patients treated with Stupp’s regimen.

## 1. Introduction

Glioblastoma, the most common malignant type of primary brain tumor in adults [1], has a poor prognosis, with an overall survival (OS) of 12–15 months [2,3]. Since the announcement of Stupp’s protocol in 2005, maximal safe resection followed by 6 weeks of concurrent chemoradiation therapy (CCRT) with adjuvant six cycles of temozolomide (TMZ) is the standard treatment. However, as most clinical trials include younger patients and those with a good performance status, a consensus regarding the optimal treatment for patients who are older or have a poor performance status has not been reached [4,5,6]. For these patients, various treatments, including standard CCRT, hypofractionated radiation therapy with or without concomitant TMZ, TMZ monotherapy, and best supportive care, are administered according to clinician judgment [7,8,9,10]. Whether the survival benefit derived from Stupp’s regimen remains in patients who are older or have a poor performance status should be further elucidated, and more prognostic factors must be identified to select the optimal treatment that balances the treatment-related survival gain and risks. Previous studies that analyzed the prognostic factors of glioblastoma mostly focused on pre-operative and tumor-related factors [6,11,12]. However, surgery can affect a patient’s performance status, morbidity, and immune and inflammatory reactions [13,14]. Here, we investigated real-world data regarding the treatment outcomes of patients with glioblastoma of various ages and statuses who were treated with Stupp’s regimen at a single tertiary institution. Furthermore, we aimed to identify the prognostic factors that could aid the decision of adjuvant treatment, including post-operative factors such as performance status and inflammatory markers.

## 2. Materials and Methods

### 2.1. Patient Selection and Evaluation of Prognostic Factors

We retrospectively reviewed the medical records of 101 patients with newly diagnosed glioblastoma who received CCRT for 6 weeks between June 2010 and January 2024. Among them, the following 21 were excluded: 17 with an isocitrate dehydrogenase (*IDH*) gene mutation or unknown *IDH* mutation status, and four who were lost to follow-up. Thus, 80 patients were included in this study. The inclusion criteria were as follows: (1) age ≥ 18 years; (2) pathological confirmation by surgical resection or stereotactic biopsy; and (3) follow-up performed using periodic contrast-enhanced magnetic resonance imaging (MRI). Patient age, sex, pre-operative tumor size (maximum diameter, cm), subventricular zone (SVZ) involvement, O (6)-methylguanine-DNA methyltransferase promoter (MGMT) methylation, extent of surgery, pre- and post-operative KPS, neutrophil-to-lymphocyte ratio (NLR), lymphocyte-to-monocyte ratio (LMR), and platelet-to-lymphocyte ratio (PLR) were investigated as prognostic factors. SVZ involvement was defined as tumor contact with the SVZ or direct ventricular invasion on pre-operative contrast-enhanced T1-weighted MRI. The extent of surgery was evaluated using post-operative MRI performed within 48 h post-operative. Gross total resection (GTR) was defined as the absence of an enhanced residual tumor on contrast-enhanced T1-weighted MRI. Patients in whom GTR was not achieved were classified as having undergone subtotal resection (STR). The pre-operative NLR, LMR, and PLR were obtained using complete blood cell counts with differential tests performed within 1 week of surgery. The post-operative KPS, NLR, LMR, and PLR evaluations were performed at 2–3 weeks post-operative, at the time of completion of post-operative care, and before the initiation of CCRT. NLR and PLR were calculated as the absolute neutrophil count and absolute platelet count divided by the absolute lymphocyte count, respectively. The LMR was computed as the ratio of the absolute lymphocyte count to the absolute monocyte count. Pre- and post-operative inflammatory markers (NLR, LMR, and PLR) were assessed in 71 of 80 patients.

### 2.2. Treatment

All glioblastoma diagnoses were pathologically confirmed by surgical resection or stereotactic biopsy. CCRT was initiated at 2–6 weeks post-operative. Temozolomide (TMZ) (75 mg/m^2^/day) was concurrently administered daily during CCRT, followed by a six-cycle maintenance regimen (150–200 mg/m^2^/day for 5 consecutive days per month) according to Stupp’s protocol [2]. Radiotherapy (RT) contouring was conducted with reference to enhanced brain MRI scans taken within 48 h post-operative and immediately before RT simulation. The gross tumor volume (GTV) was contoured to the surgical cavity and enhanced lesions on contrast-enhanced T1-weighted MRI. The clinical target volume (CTV) was generated through a margin of 1–2 cm from the GTV and included significant hypersignal lesions on T2-weighted/fluid-attenuated inversion recovery imaging. The planning target volume (PTV) was set at a 0–3 mm margin from the CTV. The median dose prescribed for the PTV was 60 Gy in 30 fractions of 2 Gy per fraction, 5 days per week.

### 2.3. Follow-Up

Clinicians evaluated the patient’s performance status, complete blood count, routine chemistry, and enhanced brain MRI at 1 month after CCRT. The response evaluation was performed using enhanced brain MRI prior to the start of the first and fourth adjuvant TMZ cycles, while periodic follow-up was performed every 3 months for 2 years and every 6 months thereafter. The response was evaluated according to the modified radiographic assessment of neuro-oncology criteria [15]. A transient enhancing lesion in the radiotherapy field within 3 months post-CCRT without clinical deterioration was considered pseudo-progression for which serial evaluations were performed. Brain perfusion computed tomography (CT) was additionally performed to differentiate between pseudo-and true progression if further evaluation was needed. Patients with increased relative cerebral blood volume and blood flow in suspicious lesions were identified as having tumor progression.

### 2.4. Statistical Analysis

The primary endpoint of this study was OS, while the secondary endpoint was progression-free survival (PFS). PFS and OS were defined as the date of the first recurrence and death or the date of the last follow-up visit from the date of surgery, respectively. PFS and OS were estimated using the Kaplan–Meier method. The log-rank test was performed to compare OS and PFS between groups. A multivariate analysis was performed using the Cox proportional hazards model for factors with values of *p* ≤ 0.05 on a log-rank test or well-known clinical significance. Areas under the curve (AUC) were calculated to estimate the prognostic efficacy of the pre- and post-operative inflammatory markers on survival (dead/alive) using a receiver operating characteristic curve. The optimal cut-off values of each variable for survival were determined using Youden’s index. Statistical significance was set at *p* < 0.05. All statistical analyses were performed using R software version 4.0.1 (R Development Core Team, Vienna, Austria).

## 3. Results

### 3.1. Clinical and Treatment Characteristics

In this study, data from 80 patients with histologically confirmed glioblastoma were retrospectively investigated. The median follow-up duration was 15.5 months (range 3.3–102.7 months). The median patient age was 60 years (range, 23−81 years). The cohort included 22 older patients (age ≥ 65 years; 27.5% of total cohort). Nineteen patients (23.8%) had poor post-operative KPS (50–60). MGMT status was identifiable in 74 patients, and MGMT methylation was detected in 39 patients (48.7%). GTR was achieved in 41 (51.2%) patients. The median interval between surgery and adjuvant CCRT was 23 days (range, 15–54 days). The median prescribed dose for RT was 6000 cGy over 30 fractions (range, 5600–6600 cGy in 28–30 fractions). Excluding two patients who exhibited aggravated neurological deficits during CCRT, 78 completed the planned 30 fractions of RT. Forty-four patients (52.5%) completed six cycles of adjuvant TMZ after CCRT. Twenty-four patients (30%) completed fewer than four cycles of adjuvant TMZ. The predominant reason for TMZ discontinuation was tumor progression, which was confirmed in 27 patients. Other reasons included infection, decreased performance status, patient refusal, and follow-up loss (in four, two, two, and one patient, respectively). Patient and treatment characteristics are shown in Table 1.

### 3.2. Survival and Prognostic Factors

**Figure 1:**
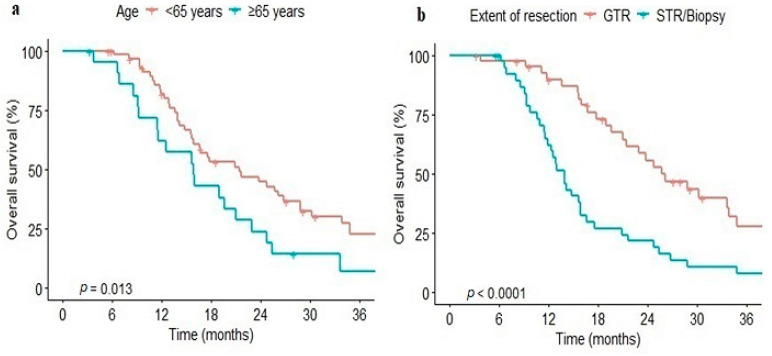
Kaplan–Meier curve of overall survival stratified by age (**a**) and extent of resection (**b**). GTR, gross total resection; STR, subtotal resection.

The median PFS of the total cohort was 8.97 months (95% confidence interval [CI], 7.43–11.9 months), while the 1- and 2-year PFS rates were 36.3% and 11.3%, respectively. The median OS was 19.0 months (95% CI, 15.7–24.8 months), while the 1- and 2-year OS rates were 76.2% and 38.8%, respectively. Sixty-four patients (80%) died, while recurrence was identified in 62 patients (77.5%) at the time of the analysis. Among the 64 deaths, medical record reviews revealed an unidentified cause for six and that four died of non-cancer-related causes, such as pulmonary thromboembolism or pneumonia. The cut-off values of pre-operative NLR, LMR, and PLR for survival were 2.0 (AUC, 0.50), 4.3 (AUC, 0.61), and 96.8 (AUC, 0.52), respectively. The post-operative NLR, LMR, and PLR cut-off values were 3.6 (AUC, 0.62), 2.5 (AUC, 0.61), and 108.5 (AUC, 0.53), respectively. In a univariate analysis, older age (≥65 years) was a negative prognostic factor for PFS (median, 8.7 vs. 9.13 months) and OS (median, 15.9 vs. 21.4 months, *p* = 0.01) compared with younger age (Figure 1). The SVZ involvement versus non-involvement group showed significantly shorter PFS (*p* = 0.003) and OS (*p* < 0.001). The GTR group showed significantly better PFS (median 11.87 months vs. 7.07 months, *p* < 0.001) and OS (median, 25.7 months vs. 13.9 months, *p* < 0.001) than the STR/biopsy group (Figure 1).

The poor post-operative KPS (50–60; *p* = 0.004) group showed significantly lower OS than the high post-operative KPS (≥70) group (median, 12.4 vs. 21.4 months, *p* = 0.003). The pre-operative KPS score was not significantly associated with OS. The higher post-operative NLR group (>3.6) showed lower OS than the lower NLR group (<3.6) with marginal statistical significance (median, 15.9 vs. 22.9 months, *p* = 0.05). The results of the univariate analysis are shown in Table 2.

In the multivariate analysis, older age (≥65 years) was an adverse factor for PFS (hazard ratio [HR], 2.58; 95% CI, 1.32−5.05; *p* = 0.005) and OS (HR, 2.19; 95% CI, 1.09−4.40; *p* = 0.026). MGMT methylation was a favorable factor for PFS (HR, 0.48; 95% CI, 0.28−0.83; *p* = 0.011) and OS (HR, 0.35; 95% CI, 0.18−0.68; *p* = 0.002), while non-GTR was an unfavorable factor for PFS (HR, 2.42; 95% CI, 1.22−4.80; *p* = 0.011) and OS (HR, 2.98; 95% CI, 1.31−6.76; *p* = 0.009). The multivariate analysis results are presented in Table 3.

### 3.3. Subgroup Analysis of Non-GTR

**Figure 2:**
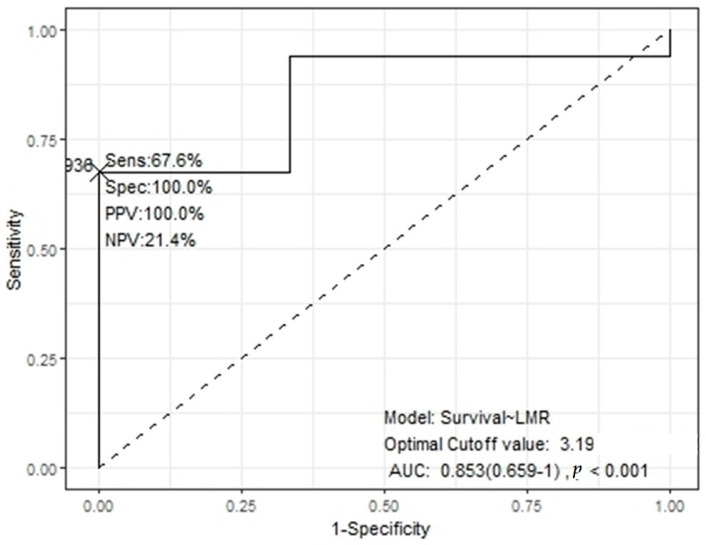
Receiver operating characteristic curve of post-operative lymphocyte-to-monocyte ratio on survival by subgroup. AUC, area under the curve; LMR, lymphocyte-to-monocyte ratio; NPV, negative predictive value; PPV, positive predictive value; Sens, sensitivity; Spec, specificity.

Based on the surgical extent being identified as a significant prognostic factor in this study, a subgroup analysis was performed of patients who underwent non-GTR. Patient age, sex, initial tumor size, SVZ involvement, MGMT methylation status, pre- and post-operative KPS, NLR, LMR, and PLR were analyzed as potential prognostic factors for PFS and OS. The pre-operative NLR, LMR, and PLR cut-off values of the subgroups were 4.4 (AUC, 0.57), 4.7 (AUC, 0.89), and 221.7 (AUC, 0.41), respectively. The post-operative NLR, LMR, and PLR cut-off values were 2.5 (AUC, 0.76), 3.2 (AUC, 0.85), and 174.4 (AUC, 0.70), respectively (Figure 2).

**Figure 3:**
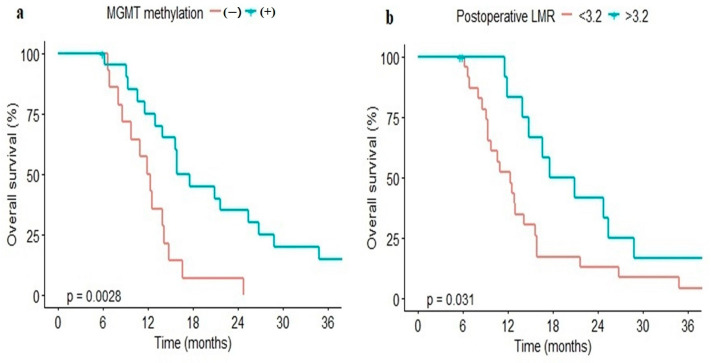
Kaplan–Meier curve of overall survival of patient treated with non-gross total resection stratified by MGMT methylation (**a**) and post-operative LMR (**b**). LMR, lymphocyte-to-monocyte ratio; MGMT, O (6)-methylguanine-DNA promoter methyltransferase.

In the univariate analysis, the MGMT methylation group showed better OS than the non-MGMT methylation group (median, 16.8 vs. 12.2 months, *p* = 0.003). The higher post-operative LMR (>3.2) group showed better PFS (median, 8.97 vs. 6.03 months, *p* = 0.003) and OS (median, 19.3 vs. 12.4 months, *p* = 0.03) than the lower LMR group (Figure 3).

Other factors such as age, sex, initial tumor size, SVZ involvement, and pre- and post-operative KPS, NLR, and PLR did not contribute significantly to PFS and OS in this subgroup. In the multivariate analysis, MGMT methylation was a favorable prognostic factor for PFS (HR, 0.39; 95% CI, 0.17−0.88; *p* = 0.024) and OS (HR, 0.20; 95% CI, 0.08−0.49; *p* < 0.001) in patients with non-GTR. A lower post-operative LMR (<3.2) was an adverse prognostic factor for PFS (HR, 0.37; 95% CI, 0.17−0.88; *p* = 0.024) and OS (HR, 0.33; 95% CI, 0.15−0.73; *p* = 0.006) (Table 4).

## 4. Discussion

This study analyzed the PFS and OS of patients with glioblastoma who received standard treatment with Stupp’s regimen [2] and identified prognostic factors for PFS and OS. The median PFS and OS were 8.97 months and 19.0 months, respectively. These results are consistent with those reported in previous studies of patients with glioblastoma who underwent 6 weeks of CCRT followed by adjuvant TMZ in which the median PFS ranged from 6.7 months to 12.7 months and median OS ranged from 14.6 months to 20 months [2,16,17]. Age, MGMT methylation, and extent of resection were independent prognostic factors for PFS and OS in this study. Age is reportedly among the most significant prognostic factors for survival in glioblastoma, as well as among patients who receive standard CCRT [18,19,20]. In this study, patients ≥ 65 years of age showed poorer PFS and OS than younger patients; in fact, all four cases of non-cancer-related deaths in this study, such as pulmonary thromboembolism or pneumonia, occurred in older patients. These results suggest that, among older patients, frailty and treatment tolerance should be evaluated to enable the selection of an adjuvant treatment that balances treatment-related benefits and risks. MGMT methylation is an independent indicator that suggests a better prognosis regardless of treatment, and survival improvement is greater with treatment of TMZ [8,21]. Consistent with previous reports, MGMT methylation was a favorable prognostic factor for PFS and OS among patients with glioblastoma who underwent 6-week CCRT followed by TMZ; its prognostic significance was also valid in a subgroup of patients who underwent non-GTR. These results suggest that treatment with Stupp’s regimen may improve survival in the presence of MGMT methylation, even among patients who undergo non-GTR in whom rapid progression and poor prognosis are expected. Numerous previous studies reported the adverse prognosis of STR versus GTR among patients with glioblastoma [22,23,24,25,26]. In this study, a subgroup analysis was performed of patients who underwent non-GTR, were expected to have early progression, and had a poor prognosis. MGMT methylation and LMR at 2–3 weeks post-operative among these patients were identified as independent prognostic factors for PFS and OS. The immune system plays an important role in tumor development and treatment response [27,28]. As a marker of systemic inflammation, LMR is reportedly a prognostic factor associated with therapeutic response and survival in several solid and hematological cancers [29,30,31,32]. In previous studies, LMR was a significant prognostic factor for OS among patients with glioma [33,34]. A meta-analysis by Wang et al. reported that a low LMR was significantly correlated with a poor OS among patients with glioblastoma [34].

Most previous studies that analyzed the prognostic implications of peripheral inflammatory markers such as NLR, LMR, and PLR in patients with glioblastoma focused on the pre-operative index [34,35,36]. In this study, to analyze the impact of post-operative inflammatory and immune responses to CCRT, we analyzed inflammatory markers pre-operatively and 2–3 weeks post-operative before CCRT. In several solid cancers, including rectal cancer, the pre-CCRT LMR is reportedly an indicator of treatment response and prognosis in patients treated with CCRT [28,37]. A study by Abe et al. of patients with stage II–III rectal cancer reported that pre-CCRT LMR was correlated with tumor size and ypT stage; in particular, a low pre-CCRT LMR < 4.0 was an adverse prognostic factor for OS [28]. Kano et al. reported that a low pre-CCRT LMR is a significant prognostic factor for DFS and OS in patients with head and neck cancer who undergo curative CCRT.

The prognostic role of the LMR at post-operative time points has also been reported. A study on breast cancer by Joanna et al. reported that post-operative LMR that measured 3–4 weeks after surgical resection is a prognostic factor for disease-free survival [38]. Lin et al. evaluated inflammatory markers at multiple time points pre- and post-operative in patients with gastric cancer to analyze their dynamic change [39]. This study found that post-operative LMR was a significant factor for OS in patients regardless of pre-operative LMR values. In our study, inflammatory markers, such as NLR, LMR, and PLR, were analyzed pre- and post-operatively. Post-operative inflammatory markers were more significant predictors of survival than pre-operative markers. A post-operative lower LMR (<3.2) did not show statistically significant results in the entire cohort; however, it showed lower PFS and OS in the subgroup of patients who underwent non-GTR. Post-operative LMR was an independent prognostic factor for PFS and OS in a multivariate analysis. This might be because, among patients with non-GTR whose post-operative residual tumor burden is high, the host immune reaction may have a greater impact on tumor cell killing. In a previous study of hilar cholangiocarcinoma by Lin et al., LMR positively correlated with CD3+ T-cell tumor infiltration, and a low CD3+ T-cell infiltration was an adverse prognostic factor for OS [40]. In previous studies, high NLR and PLR were suggested as adverse prognostic factors for the survival of patients with glioblastoma [41,42,43], but our study did not show significant results for these factors in all cohorts and subgroups. The points at which inflammatory index becomes a more significant prognostic factor for patients with glioblastoma, and whether comprehensive interpretation of various inflammatory indices could enhance the prognostic relevance, should be clarified through further studies. In addition, the prognostic significance of inflammatory markers pre-CCRT but post-operative requires further validation. More individualized therapies that balance treatment benefits and risks through sophisticated predictions of treatment response and prognosis using these markers should be considered in the future.

This study has several limitations. First, it was a retrospective analysis of patients who received treatment at a single institution; therefore, selection bias may be inherent. Second, a relatively low number of patients who were older or had a poor KPS were included. Third, inflammatory markers such as NLR and LMR were not assessed at the same time point across all patients, i.e., variations within 1 week were observed, which may have affected the results. These could be sources of bias for observational study, and caution should be exercised when interpreting the results. Further studies are needed to determine the most meaningful measurement time points to establish the prognostic value of the post-operative inflammatory index. Fourth, information on molecular markers such as telomerase reverse transcriptase or alpha thalassemia/mental retardation syndrome X-linked mutations that may affect prognosis could be identified in only a few patients; therefore, an analysis of these factors was not performed. Despite these limitations, this study identified older age (>65 years), MGMT non-methylation, and non-GTR as adverse prognostic factors in patients with glioblastoma who underwent standard treatment according to Stupp’s protocol. In addition, this study suggests that a lower post-operative LMR is a poor prognostic factor in patients with non-GTR. Deintensified therapies, such as hypofractionated radiation therapy and TMZ alone, may need to be considered for patients expected to have poor survival outcomes owing to these unfavorable prognostic factors, considering the potential toxicity of therapy, quality of life, and treatment convenience. Further studies are needed to validate the correlation between the post-operative inflammatory index, response to CCRT, and survival, as well as to identify the most meaningful measuring time point for the prognostic role of the post-operative inflammatory index.

## 5. Conclusions

This study investigated the outcomes of patients with glioblastoma who received treatment using Stupp’s protocol in a real-world clinical setting. Our findings confirmed that age, MGMT methylation, and extent of surgery, which are known significant factors in the survival of patients with glioblastoma, were valid prognosticators in patients who received treatment with Stupp’s regimen. MGMT methylation and post-operative LMR were independent prognostic factors for PFS and OS in patients who underwent non-GTR. Our findings suggest that the prognostic implications of post-operative inflammatory markers on the treatment response and survival of patients who received treatment with Stupp’s regimen should be validated in further studies.

## Figures and Tables

**Table 1:** Patient and treatment characteristics.

Characteristics	N. (%) (Total, N = 80)
**Age**	Median, 60 years (range, 23–81 years)
**Gender**	
Male	44 (55%)
Female	36 (45%)
**Pre-operative KPS**	
40–60	15 (18.8%)
70–100	61 (76.2%)
Unknown	4 (5%)
**Post-operative KPS**	
50–60	19 (23.8%)
70–100	61 (76.2%)
**Comorbidities at pre-CCRT**	
Hemiplegia	14 (17.5%)
Diabetes	9 (11.3%)
Cerebrovascular disease	4 (5%)
Pulmonary disease	3 (3.8%)
Alcoholic dementia	2 (2.5%)
Peripheral vascular disease	1 (1.3%)
Congestive heart failure	1 (1.3%)
Hepatitis	1 (1.3%)
Ulcer disease	1 (1.3%)
**Subventricular zone involvement**	
Yes	46 (57.5%)
No	32 (40%)
Unknown	2 (2.5%)
**MGMT methylation**	
Yes	39 (48.7%)
No	35 (43.8%)
Unknown	6 (7.5%)
**Ki-67 index (%)**	Median, 25.5 (range, 2.8–78.7)
**Extent of resection**	
Gross total resection	41 (51.2%)
Subtotal resection	32 (40%)
Biopsy	7 (8.8%)
**Radiotherapy**	Median, 60 Gy/30 fractions (range, 56–66 Gy/28–30 fractions)
**Completion of planned radiotherapy**	78 (97.5%)
**Adjuvant temozolomide cycle**	
0–3 cycles	24 (30%)
4–5 cycles	12 (15%)
6 cycles	44 (52.5%)

CCRT, concurrent chemoradiation therapy; KPS, Karnofsky Performance Scale; MGMT, O (6)-methylguanine DNA methyltransferase; N, number.

**Table 2:** Univariate analysis of prognostic factors for progression-free and overall survival.

Factor	N (N = 80)	Median PFS (Months)	*p*-Value	Median OS (Months)	*p*-Value
**Age (years)**			0.02 *		0.01 *
<65	58	9.13		21.4	
≥65	22	8.70		15.9	
**Sex**			0.1		0.5
Male	44	9.2		19.6	
Female	36	6.1		17.8	
**Tumor size (cm)**			0.5		0.9
<4	29	8.33		21.1	
≥4	47	9.10		17.6	
**SVZ involvement**			<0.001 *		<0.001 *
(−)	32	11.87		28.9	
(+)	46	7.37		14.2	
**MGMT** **methylation**			0.2		0.1
(−)	35	8.37		16.6	
(+)	39	11.4		21.4	
**Extent of** **resection**			<0.001 *		<0.001 *
GTR	41	11.87		25.7	
STR/biopsy	39	7.07		13.9	
**Pre-operative KPS**					0.7
≤60	15	10.77	0.8	21.4	
70–100	61	8.77		19.0	
**Post-operative KPS**			0.03 *		0.003 *
50–60	19	7.07		12.4	
70–100	61	9.2		21.4	
**Pre-operative NLR**			0.9		0.9
≤2.0	23	9.2		21.1	
>2.0	48	8.57		16.0	
**Pre-operative LMR**			0.6		0.2
<4.3		8.37		15.7	
>4.3		9.7		21.6	
**Pre-operative PLR**			0.1		0.5
<96.8	11	7.13		21.1	
>96.8	60	9.10		17.6	
**Post-operative NLR**			0.3		0.05 *
<3.6	37	8.7		22.9	
>3.6	34	9.2		15.9	
**Post-operative LMR**			0.3		0.2
<2.5	28	9.2		15.9	
>2.5	43	8.7		19.0	
**Post-operative PLR**			0.4		0.8
<108.5	11	12.8		22.9	
>108.5	60	8.7		16.8	

GTR, gross total resection; KPS, Karnofsky Performance Scale; LMR, lymphocyte-to-monocyte ratio; MGMT, O (6)-methylguanine-DNA methyltransferase; N, number; NLR, neutrophil-to-lymphocyte ratio; OS, overall survival; PFS, progression-free survival; PLR, platelet-to-lymphocyte ratio; STR, subtotal resection; SVZ, subventricular zone. * Statistically significant.

**Table 3:** Multivariate analysis of prognostic factors for progression-free and overall survival.

Factor		PFS		OS	
		HR (95% CI)	*p*-Value	HR (95% CI)	*p*-Value
Age (years)	<65/≥65	2.58 (1.32–5.05)	0.005 *	2.19 (1.09–4.40)	0.026 *
SVZ involvement	(−)/(+)	1.53 (0.81–2.89)	0.18	1.43 (0.68–0.98)	0.337
MGMT methylation	(−)/(+)	0.48 (0.28–0.83)	0.009 *	0.35 (0.18–0.68)	0.002 *
Extent of resection	GTR/non-GTR	2.42 (1.22–4.80)	0.011 *	2.98 (1.31–6.76)	0.009 *
Post-operative KPS	50–60/70–100	1.05 (0.49–2.23)	0.893	1.05 (0.44–2.54)	0.897
Post-operative NLR	<3.6/>3.6	-	-	1.58 (0.84–2.96)	0.154

GTR, gross total resection; KPS, Karnofsky Performance Scale; MGMT, O (6)-methylguanine-DNA methyltransferase promoter; NLR, neutrophil-to-lymphocyte ratio; OS, overall survival; PFS, progression-free survival; SVZ, subventricular zone. * Statistically significant.

**Table 4:** Multivariate analysis of prognostic factors for progression-free and overall survival for patients with non-GTR.

Factor		PFS			OS	
		HR (95% CI)		*p*-Value	HR (95% CI)	*p*-Value
Age (years)	<65/≥65	2.06 (0.88–4.81)		0.09	2.14 (0.95–4.81)	0.06
MGMT methylation	(−)/(+)	0.39 (0.17–0.88)		0.024 *	0.20 (0.08–0.49)	<0.001 *
Post-operative LMR	<3.2/>3.2	0.37	(0.17–0.81)	0.014 *	0.33 (0.15–0.73)	0.006 *

LMR, lymphocyte-to-monocyte ratio; MGMT, O (6)-methylguanine-DNA methyltransferase promoter; OS, overall survival; PFS, progression-free survival. * Statistically significant.

## Data Availability

The original contributions presented in this study are included in the article. Further inquiries can be directed to the corresponding author.

## Abbreviations

The following abbreviations are used in this manuscript:

CCRT	Concurrent chemoradiation
CI	Confidence interval
CT	Computed tomography
CTV	Clinical target volume
GTR	Gross total resection
GTV	Gross tumor volume
HR	Hazard ratio
KPS	Karnofsky Performance Scale
LMR	Lymphocyte-to-monocyte ratio
MGMT	O (6)-methylguanine-DNA methyltransferase promotor
MRI	magnetic resonance imaging
NLR	Neutrophil-to-lymphocyte ratio
OS	Overall survival
PFS	Progression-free survival
PLR	Platelet-to-lymphocyte ratio
PTV	Planning target volume
RT	Radiotherapy
STR	Subtotal resection
SVZ	Subventricular zone
TMZ	Temozolomide
